# Supplementary material for: Modeling glioblastoma heterogeneity as a dynamic network of cell states
Source: Mol Syst Biol. 2021 Sep 16;17(9):e10105. doi: 10.15252/msb.202010105 (PMC8444284; doi:10.15252/msb.202010105)
Supplement: Supplementary file 6 — Source Data for Figure 5 [file MSB-17-e10105-s004.zip › Figure5A_sourcedata/GSEA_3017/hallmarks_stateE.GseaPreranked.1621934677158/HALLMARK_APOPTOSIS.html]

Details for gene set HALLMARK\_APOPTOSIS[GSEA]

|  || Dataset | state63017 |
| Phenotype | NoPhenotypeAvailable |
| Upregulated in class | na\_pos |
| GeneSet | HALLMARK\_APOPTOSIS |
| Enrichment Score (ES) | 0.36341763 |
| Normalized Enrichment Score (NES) | 1.8834432 |
| Nominal p-value | 0.0 |
| FDR q-value | 0.010420741 |
| FWER p-Value | 0.019 |
Table: GSEA Results Summary

  

Fig 1: Enrichment plot: HALLMARK\_APOPTOSIS      
 Profile of the Running ES Score & Positions of GeneSet Members on the Rank Ordered List

  

| PROBE | GENE SYMBOL | GENE\_TITLE | RANK IN GENE LIST | RANK METRIC SCORE | RUNNING ES | CORE ENRICHMENT || 1 | ANXA1 |  |  | 12 | 0.826 | 0.0506 | Yes |
| 2 | CD44 |  |  | 14 | 0.806 | 0.1115 | Yes |
| 3 | LGALS3 |  |  | 25 | 0.697 | 0.1543 | Yes |
| 4 | IFITM3 |  |  | 26 | 0.682 | 0.2068 | Yes |
| 5 | NEDD9 |  |  | 38 | 0.590 | 0.2403 | Yes |
| 6 | EMP1 |  |  | 40 | 0.584 | 0.2842 | Yes |
| 7 | MMP2 |  |  | 57 | 0.503 | 0.3055 | Yes |
| 8 | ATF3 |  |  | 101 | 0.397 | 0.2894 | Yes |
| 9 | TIMP3 |  |  | 106 | 0.391 | 0.3152 | Yes |
| 10 | CLU |  |  | 119 | 0.364 | 0.3302 | Yes |
| 11 | BNIP3L |  |  | 142 | 0.341 | 0.3326 | Yes |
| 12 | RHOB |  |  | 148 | 0.335 | 0.3530 | Yes |
| 13 | JUN |  |  | 172 | 0.316 | 0.3524 | Yes |
| 14 | TGFB2 |  |  | 189 | 0.303 | 0.3584 | Yes |
| 15 | SAT1 |  |  | 206 | 0.291 | 0.3634 | Yes |
| 16 | TNFRSF12A |  |  | 262 | 0.257 | 0.3235 | No |
| 17 | GSR |  |  | 349 | -0.268 | 0.2509 | No |
| 18 | TXNIP |  |  | 404 | -0.284 | 0.2142 | No |
| 19 | CDK2 |  |  | 422 | -0.290 | 0.2181 | No |
| 20 | VDAC2 |  |  | 492 | -0.311 | 0.1672 | No |
| 21 | BTG3 |  |  | 615 | -0.365 | 0.0629 | No |
| 22 | SLC20A1 |  |  | 717 | -0.444 | -0.0124 | No |
| 23 | BRCA1 |  |  | 846 | -0.691 | -0.0981 | No |
| 24 | HMGB2 |  |  | 937 | -1.238 | -0.1004 | No |
| 25 | TOP2A |  |  | 942 | -1.417 | 0.0043 | No |
Table: GSEA details [plain text format]

  

Fig 2: HALLMARK\_APOPTOSIS: Random ES distribution      
 Gene set null distribution of ES for **HALLMARK\_APOPTOSIS**
